# The Effects of Concurrent Strength and Endurance Training on Physical Fitness and Athletic Performance in Youth: A Systematic Review and Meta-Analysis

**DOI:** 10.3389/fphys.2018.01057

**Published:** 2018-08-07

**Authors:** Martijn Gäbler, Olaf Prieske, Tibor Hortobágyi, Urs Granacher

**Affiliations:** ^1^Division of Training and Movement Sciences, Research Focus Cognition Sciences, Faculty of Human Sciences, University of Potsdam, Potsdam, Germany; ^2^Center for Human Movement Sciences, University Medical Center Groningen, University of Groningen, Groningen, Netherlands

**Keywords:** child, adolescent, muscle strength, cardiorespiratory fitness, physical conditioning human, resistance training, youth sports

## Abstract

Combining training of muscle strength and cardiorespiratory fitness within a training cycle could increase athletic performance more than single-mode training. However, the physiological effects produced by each training modality could also interfere with each other, improving athletic performance less than single-mode training. Because anthropometric, physiological, and biomechanical differences between young and adult athletes can affect the responses to exercise training, young athletes might respond differently to concurrent training (CT) compared with adults. Thus, the aim of the present systematic review with meta-analysis was to determine the effects of concurrent strength and endurance training on selected physical fitness components and athletic performance in youth. A systematic literature search of PubMed and Web of Science identified 886 records. The studies included in the analyses examined children (girls age 6–11 years, boys age 6–13 years) or adolescents (girls age 12–18 years, boys age 14–18 years), compared CT with single-mode endurance (ET) or strength training (ST), and reported at least one strength/power—(e.g., jump height), endurance—(e.g., peak V°O_2_, exercise economy), or performance-related (e.g., time trial) outcome. We calculated weighted standardized mean differences (SMDs). CT compared to ET produced small effects in favor of CT on athletic performance (*n* = 11 studies, SMD = 0.41, *p* = 0.04) and trivial effects on cardiorespiratory endurance (*n* = 4 studies, SMD = 0.04, *p* = 0.86) and exercise economy (*n* = 5 studies, SMD = 0.16, *p* = 0.49) in young athletes. A sub-analysis of chronological age revealed a trend toward larger effects of CT vs. ET on athletic performance in adolescents (SMD = 0.52) compared with children (SMD = 0.17). CT compared with ST had small effects in favor of CT on muscle power (*n* = 4 studies, SMD = 0.23, *p* = 0.04). In conclusion, CT is more effective than single-mode ET or ST in improving selected measures of physical fitness and athletic performance in youth. Specifically, CT compared with ET improved athletic performance in children and particularly adolescents. Finally, CT was more effective than ST in improving muscle power in youth.

## Introduction

Physical activity promotes motor development and physical fitness in youth. The World Health Organization recommends at least 60 min of moderate- to vigorous-intensity physical activity daily in youth aged 5–17 years. Most of the physical activity should be aerobic with additional muscle strengthening exercises conducted at least three times per week (WHO, 2010). Thus, the general youth population should regularly perform endurance and strength exercises. While physical activity promotes motor development in youth, young athletes may specifically benefit from endurance training (ET) and strength training (ST) during long-term development of sport-specific athletic performance. Indeed, high levels of muscular strength and aerobic endurance are key determinants of success in many sports (Baar, 2014; Bompa and Buzzichelli, 2015). According to the concept of training specificity (Häkkinen et al., 1989; Behm, 1995), ST improves muscular strength and ET improves cardiorespiratory endurance.

To increase athletic performance, athletes and coaches seek ways to optimize training and minimize risks for injury. A promising way to increase performance is to train both muscle strength and cardiorespiratory fitness within a training cycle [i.e., concurrent training (CT)]. CT could potentiate the individual effects produced by ET and ST and increase athletic performance more than training ET and ST alone. A favorable interaction between ST and ET may reduce time spent on ST and ET and increase time for recovery or training for sport-specific skills. Indeed, CT compared with single-mode ET may produce larger performance improvements in time trials in runners and cyclists (Rønnestad and Mujika, 2014). In addition, when elite cyclists combined cycling and lower limb progressive resistance training, CT improved mean power output during a 45 min cycle-ergometer test more (Δ: 26.4 W, 8.4%) than did ET (Δ: 11.5 W, 3.7%) (Aagaard et al., 2011).

However, ST and ET could also interfere with each other (Docherty and Sporer, 2000) and produce inferior gains in muscular strength compared with ST, resulting in an “interference effect” (Hickson, 1980; Wilson et al., 2012). Interference occurs when strength and endurance stimuli both target peripheral (i.e., muscular) adaptations (e.g., hypertrophy vs. muscle capillarisation) (Docherty and Sporer, 2000) and a meta-analysis confirmed the CT-related “interference hypothesis” (Wilson et al., 2012). That is, ST alone compared to CT produced larger improvements in muscle strength (within group standardized mean differences [SMD]: 1.76 vs. 1.44), muscle hypertrophy (within group SMD: 1.23 vs. 0.85), and muscle power (within group SMD: 0.91 vs. 0.55).

Current theories on the potentiating or interfering effects in CT have been derived from data in adult humans and animals. Because anthropometric, physiological, and biomechanical differences between youth and adults can affect the responses to exercise training, youth compared with adults might respond differently to CT. That is, the physiological processes associated with growth and maturation make the application of adult data to children untenable. For instance, Spurrs et al. (2003) found positive effects of CT compared to ET on 3 km performance {CT: −10 s [1.6%]; ET: −3 s [0.5%]} and running economy at running velocities above 12 km/h (CT: 4–7%; ET: < 1%) in 25-year-old distance runners, whereas Bluett et al. (2015) reported a slight decrease in 3 km performance in 10–13 year old distance runners in the CT group (Δ: 6 s, 0.8%), but a slight improvement in the ET group (Δ: −17 s, 2.1%). Further, ST designed to induce hypertrophy in adults (Fleck and Kraemer, 2014) failed to produce hypertrophy in prepubescent children (Ozmun et al., 1994; Granacher et al., 2011). In addition, a 10 week machine-based ST using sub-maximal intensities (70–80% of the 1-repetition maximum [1RM]) increased lower-limb muscle strength but not quadriceps cross-sectional area as measured with magnetic resonance imaging in prepubertal children (Granacher et al., 2011). The apparent inability of children's muscles to hypertrophy following training is attributed to low levels of androgens (Viru et al., 1999; Legerlotz et al., 2016).

Given the anthropometric, physiological, and biomechanical differences between youth and adults and the need to optimize the training stimulus, the present review with meta-analysis aimed to determine whether CT compared with single-mode ET and ST would produce a potentiating or interfering effect in children and adolescents. Specifically, we compared the effects of CT and ET on endurance-related outcomes (cardiorespiratory endurance, exercise economy) and on athletic performance (e.g., time trials) and the effects of CT and ST on strength-related outcomes (maximum muscle strength, muscle power, muscle hypertrophy). We formulated three hypotheses based on previous work. First, given the role of muscle strength in youth physical development and sports (Lloyd and Oliver, 2012; Faigenbaum et al., 2016), we hypothesized that CT is more effective than single-mode ET in improving athletic performance as assessed by time trials. Second, we hypothesized that CT compared to single-mode training results in larger improvements in physical fitness because CT results in adaptations of the muscular and cardiorespiratory systems that are both related to physical fitness outcomes. Third, we hypothesized that CT-related interference effects in strength adaptations are age-dependent and present in adolescents but not in children because prepubescent children appear not to have the physiological basis for training-induced muscle hypertrophy.

## Methods

The systematic literature search and meta-analysis was performed in accordance with the recommendations of the Preferred Reporting Items for Systematic reviews and Meta-Analyses (PRISMA) statement (Moher et al., 2010).

### Literature search

The electronic databases PubMed and Web of Science were consulted from 1980 until June 7th 2018 using the following Boolean search syntax: “(youth OR young OR children OR adolescents OR pubertal OR boys OR girls OR school) AND (athlete OR sport OR trained) AND (training OR exercise) AND (concurrent OR combined OR combination OR additional) AND (strength OR resistance OR endurance OR aerobic) NOT (elderly OR student OR college OR patient OR disease OR syndrome OR (cerebral palsy) OR injury OR sedentary OR obese OR animals OR supplementation OR validity).” Where available, we applied filters to limited the output of the search according to the age of participants (Child: 6–12 years; Adolescent: 13–18 years), language (English), article type (no review), and research areas (sport sciences or physiology). Additionally, the reference lists of relevant studies were screened.

### Eligibility criteria

We formed eligibility criteria using the PICOS (Population, Interventions, Comparators, Outcomes, Study design) approach (University of York, Centre for Reviews and Dissemination., 2009). Studies were found eligible for inclusion in the meta-analysis when participants where healthy children or adolescents age 6–18 years. Because biological age is not often reported in studies (Lesinski et al., 2016), participants were categorized based on their chronological age according to Faigenbaum et al. (2009) as children (boys age 6–13 y and girls age 6–11 y) or adolescents (boys age 14–18 y and girls age 12–18 y). Furthermore, we used the definition proposed by Williams (2016) to distinct young athletes from non-athletic youth, namely: “a child or adolescent who is still growing and maturing toward adulthood and who systematically trains (> once per week) and competes (>1-year competition history) in at least one specific sport.” With respect to the intervention, studies needed to have applied a CT protocol to at least one group in the study. Furthermore, at least one active control group was required that followed single-mode ET or ST to function as a comparator. For athletes, this meant that a major part of their training consisted of either ET or ST. Studies with two or more different concurrent training protocols, but without a single-mode training group were included in the qualitative analysis of the review but not in the meta-analysis. Means and standard deviations of one or more of the following outcomes had to be reported for all groups before and after intervention: measures of maximum muscle strength, muscle power, muscle hypertrophy, cardiorespiratory endurance, exercise economy, and athletic performance (see also Table 1). We defined athletic performance as a sport-specific competitive outcome (e.g., time trial, ball kicking velocity).

**Table 1:** Preferred and alternative outcomes for each outcome measure.

**Category**	**Preferred outcome**	**Alternative outcome(s)**
Maximum muscle strength	1 repetition maximum	>1 repetition maximum Dynamometry
Muscle power	Countermovement jump	Other jump height Dynamometry
Muscle hypertrophy	Muscle cross-sectional area	Muscle mass Muscle thickness
Cardiorespiratory endurance	Peak V°O_2_	V°O_2_max Estimated V°O_2_max
Exercise economy	V°O_2_ at submaximal velocity	–
Athletic performance	Relevant sport specific outcome (e.g., time trial, kicking / throwing velocity)	–

The selection process started with the removal of duplicate studies, followed by the screening of titles, abstracts and eventually full texts of the respective studies.

### Data collection

Pre- and post-test means and standard deviations (SDs) were preferably collected from numerical data reported in publications. Authors were contacted in case of unreported data. When authors did not respond, means and SDs were estimated from figures using GetData Graph Digitizer (http://www.getdata-graph-digitizer.com/). Ultimately, SDs were deduced by estimating post-test SD from pre-test SD. Outcomes were excluded when crucial data were still missing.

If more than one outcome measure was reported for a certain variable, only one outcome was included in the analyses to prevent bias. As a general remark, easily administered field tests were preferred over more sophisticated lab measures for the sake of homogeneity, because only few studies reported lab measures. An overview of preferred and alternative outcomes can be found in Table 1.

### Risk of bias assessment

Heterogeneity between studies was assessed using I^2^ percentages for each outcome and interpreted according to Higgins et al. (2003) as low (>25%), moderate (>50%), or high (>75%). The risk of bias and methodological quality of the included studies were further quantified through the Physiotherapy Evidence Database (PEDro) scale (Maher et al., 2003). The PEDro scale consists of 11 dichotomous questions of which 10 are evaluated. Scoring ranges from 0 to 10 where a higher score indicates a lower risk of bias. A score ≥ 6 is indicative of a high study quality. A score ≥ 4 indicates a fair study quality.

### Statistical analyses

For each study, between-group standardized mean differences (SMD) were calculated for post-test mean values (m) and corrected for sample size (N) according to Hedges and Olkin (1985) $\left(SMD_{between}=\frac{m_{1i-}m_{2i}}{\;s_{i}}\cdot \left(1-\frac{3}{4N-9}\right)\right)$. SMDs were multiplied by−1 for measures where an improvement in performance was indicated by a negative change (e.g., time trials). Further analyses were performed in Review Manager 5.3.5 (Copenhagen: The Nordic Cochrane Centre, The Cochrane Collaboration, 2014). To compare the effects of CT to the effects of single-mode ET and ST on different outcome measures, SMDs where weighted with respect to their standard errors and aggregated to compute the overall SMD using a random effects model. Overall SMDs were interpreted according to Cohen (1988) as trivial (SMD < 0.2), small (0.2 ≤ SMD < 0.5), moderate (0.5 ≤ SMD < 0.8), or large (0.8 ≤ SMD). Chi-squared (χ^2^) statistics were calculated to determine differences in outcomes between sub-groups. See Deeks and Higgins (2010) for a more detailed description on formulae.

Within group SMDs were calculated as $\left(SMDwithin=\frac{m_{post-}m_{pre}}{\;sd_{pre}}\right)$. Relationships between within group SMDs of outcome measures and group characteristics were quantified by Pearson correlation coefficients.

An α of 0.05 was used to determine statistical significance.

## Results

**Figure 1:**
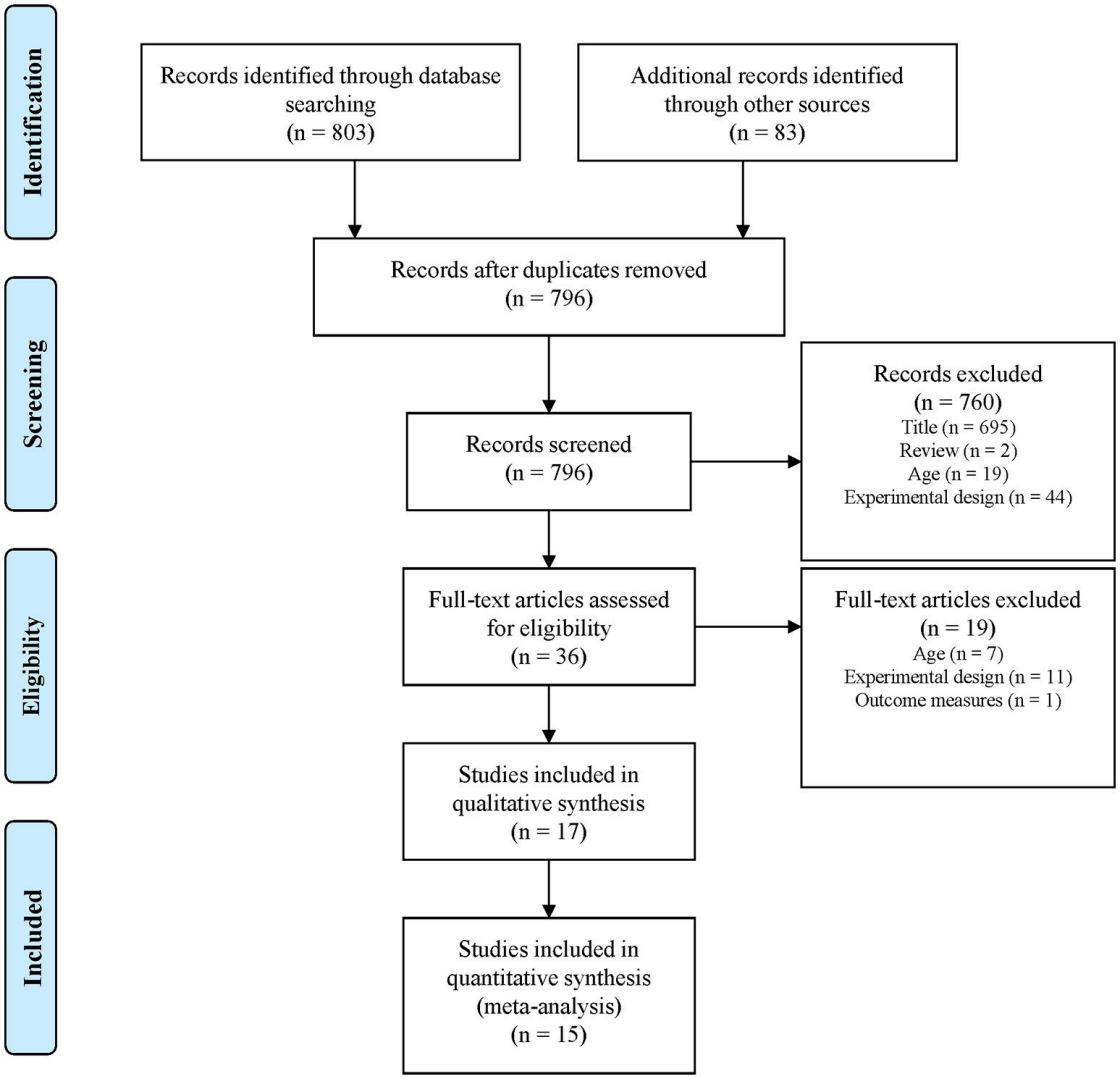
Flow diagram describing the study selection process. Adapted from Moher et al. (2010).

The systematic search identified 886 records. Figure 1 shows the article selection process. Fifteen studies were eligible for inclusion in the meta-analysis with a total of 33 training groups, of which eighteen, eleven, and four groups were categorized as CT, ET, and ST, respectively. Eleven studies examined young athletes (swimming, running, rowing) and compared CT with ET and four studies involved non-athletic youth and compared CT with ET. In total, the number of participants was 518 (268 male, 250 female). Mean ages ranged from 10.7 to 18.2 (median = 14.1) years and fourteen training groups were classified as children and nineteen as adolescents. Two additional records (Enright et al., 2015; Makhlouf et al., 2016) were not eligible for inclusion in the meta-analysis, but were included in the qualitative analysis. Table 2 characterizes the populations and training programs and shows the quality assessment (PEDro) scores (range: 3 to 7, median = 4).

**Table 2:** Study characteristics.

**Author Name, year**	**Sport**	**Tr grp**	**N (m/f)**	**B. Age**	**C. Age**	**Endurance training**					**Strength training**							**P E D r o**
						**Contents**	**W**	**F**	**Time or distance**	**Intensity**	**Contents**	**W**	**F**	**S**	**Reps**	**Rest**	**Intensity %RM**	
Aspenes et al., 2009	Swimming	CT	11 (6/5)	–	17.5 ± 2.9	Interval swimming	11	2	4 x 4 min 3 x 3 min	90–95% HF_max_ 60–75% HF_max_	Machine	11	2	3	5	2–5	5 RM	3
		ET	9 (2/7)	–	15.9 ± 1.1	Interval swimming	11	2	4 x 4 min 3 x 3 min	90–95% HF_max_ 60–75% HF_max_								
Amaro et al., 2017	Swimming	CT	7 (7/0)	–	12.7 ± 0.8	Swimming	10	6			Power, core training	6	2	3	6–18	40–90 s	–	7
		CT	7 (7/0)	–	12.7 ± 0.8	Swimming	10	6			Power, core training	6	2	3	10-25s	40–100 s	–	
		ET	7 (7/0)	–	12.6 ± 0.8	Swimming	10	6	4.1 km									
Blagrove et al., 2018	Running	CT	9 (4/5)	3.1	16.5 ± 1.1	Running	10		151 min/week	High volume, low intensity	Plyometric and free weight	10	2	2–4	6–15	90–180 s	–	4
		ET	9 (4/5)	3.9	17.6 ± 1.2	Running	10		213 min/week	High volume, low intensity								
Bluett et al., 2015	Running	CT	6 (3/3)	–	10–13	Continous and interval running	10	2	30 min/300 m 800 m	60–100% of maximal effort	Machine	10	1	3-4	10-12	2	70–75% 1RM	4
		ET	6 (3/3)	–	10–13	Continous and interval running	10	2	30 min/300 m 800 m	60–100% of maximal effort								
Carlsson et al., 2017	Cross country ski	CT	14 (7/7)	–	18.5 ± 0.9	Regular training					Free weight, body weight	6	2	2–3	30–90 s	60 s	6–8RM/ 60–85% BW	4
		ET	19 (9/10)	–	18.1± 0.9	Ski-ergometer	6	2	40 min	90–100% HF_max_								
Egan-Shuttler et al., 2017	Rowing	CT	8 (8/0)	–	16 ± 0.8	Rowing	4	3	30 min		Plyometric	4	3	2–5	5–20		–	4
		ET	8 (8/0)	–	16 ± 0.6	Cycling + Rowing	4	3	2 x 30 min	Low intensity								
Garrido et al., 2010	Swimming	CT	12 (8/4)	1–2	12.0 ± 0.8	Swimming	8	6	1.5 h	70% VO2_max_	Free weight	8	2	2–3	6–8	2	50–75%/6RM	4
		ET	11 (6/5)	1–2	12.2 ± 0.8	Swimming	8	6	1.5 h	70% VO2max								
Girold et al., 2007	Swimming	CT	7 (4/3)	–	16.5 ± 2.5	Swimming + cycling	12		8.3 h/week	60–70% HF_max_	Machine	12	1	3	6	2	80–90% 1RM	4
		CT	7 (3/4)	–	16.5 ± 2.5	Swimming + cycling	12		8.3 h/w	60–70% HF_max_	Resisted swimming	12	1	2	3	30 s		
		ET	7 (3/4)	–	16.5 ± 1.5	Swimming + cycling	12		10.8 h/w	60–70% HF_max_								
Mikkola et al., 2007	Running	CT	13 (9/4)	–	17.3 ± 0.9	Running training	8		7.2 h/w	–	High velocity	8	1	2–3	6–10	–	No or low load	4
		ET	12 (9/3)	–	17.3 ± 0.5	Running training	8		8.5 h/w	–								
Potdevin et al., 2011	Swimming	CT	12 (5/7)	3–4	14.3 ± 0.2	Swimming	6	1	5.5 h/w	–	Plyometric	6	2	1–8	1–16	–	BW	4
		ET	11 (5/6)	3– 4	14.1 ± 0.2	Swimming	6	1	5.5 h/w	–								
Weston et al., 2015	Swimming	CT	10 (5/6)	–	15.7 ± 1.2	Swimming	12	8	8 h/w	–	Core training	6	2	2	60 s	60 s	BW	3
		ET	10 (5/6)	–	16.7 ± 0.9	Swimming	12	8	8 h/w	–								
Alves et al., 2016	–	CT	38 (21/17)	–	11.0 ± 0.5	20 m shuttle run	8	2	–	≤ Estimated 75% VȮ_2_max	Ball throw; jump, sprint	8	2	1–4	4–8	–	–	5
		CT	45 (21/24)	–	10.8 ± 0.5	20 m shuttle run	8	2	–	≤ Estimated 75% VȮ_2_max	Ball throw; jump, sprint	8	2	1–4	4–8	–	–	
		ST	41 (19/22)	–	10.8 ± 0.4						Ball throw; jump, sprint	8	2	1–4	4–8	–	–	
Marta et al., 2013	–	CT	45 (21/24)	1–2	10.7 ± 0.5	20 m shuttle run	8	2	–	≤ Estimated 75% VȮ_2_max	Ball throw; jump, sprint	8	2	1–5	3–8	1–2	–	5
		ST	41 (19/22)	1–2	10.7 ± 0.4						Ball throw; jump, sprint	8	2	1–5	3–8	1–2	–	
Santos et al., 2012	–	CT	25 (0/25)	–	13.5 ± 1.0	20 m shuttle run	8	2	–	≤ Estimated 75% VȮ_2_max	Ball throw; jump, sprint	8	2	1–5	3–8	–	–	5
		ST	21 (0/21)	–	13.5 ± 1.0						Ball throw; jump, sprint	8	2	1–5	3–8	–	–	
Santos et al., 2012	–	CT	15 (15/0)	–	13.3 ± 1.0	20 m shuttle run	8	2	–	≤ Estimated 75% VȮ_2_max	Ball throw; jump, sprint	8	2	1–5	3–8	–	–	6
		ST	15 (15/0)	–	13.3 ± 1.0						Ball throw; jump, sprint	8	2	1–5	3–8	–	–	
Enright et al., 2015	Soccer	CT	8 (0/8)	–	17.3 ± 1.6	Soccer specific ET	5	2	3.2	–	Free-weight and body weight	5	2	3–4	6–8	–	85%/ 60–80% 1RM	4
		CT	7 (0/7)	–	17.3 ± 1.6	Soccer specific ET	5	4	3.2	–	Free-weight and body weight	5	2	3–4	6–8	–	85%/ 60–80% 1RM	
Makhlouf et al., 2016	Soccer	CT	14 (14/0)	–	13.7 ± 0.5	Interval running	12	4	15 s	110–120% of aerobic test	23 whole body	12	2	3	5–15	–	Body-weight	4
		CT	15 (15/0)	–	13.7 ± 0.5	Interval running	12	4	15 s	110–120% of aerobic test	23 whole body	12	2	3	5–15	–	Body-weight	

*Tr grp, training group; B. Age, biological age; C, chronological age; W, number of weeks; F, training frequency per week; S, number of sets per exercise; Reps, number of repetitions per set; 1RM, one repetition maximum; HF, heart frequency; BW, Body weight (as resistance)*.

**Figure 2:**
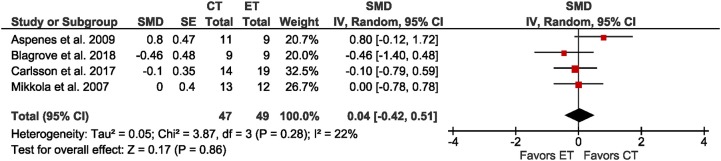
Forest plot for the outcome cardiorespiratory endurance in the comparison between singular endurance training (ET) and concurrent strength and endurance training (CT).

**Figure 3:**
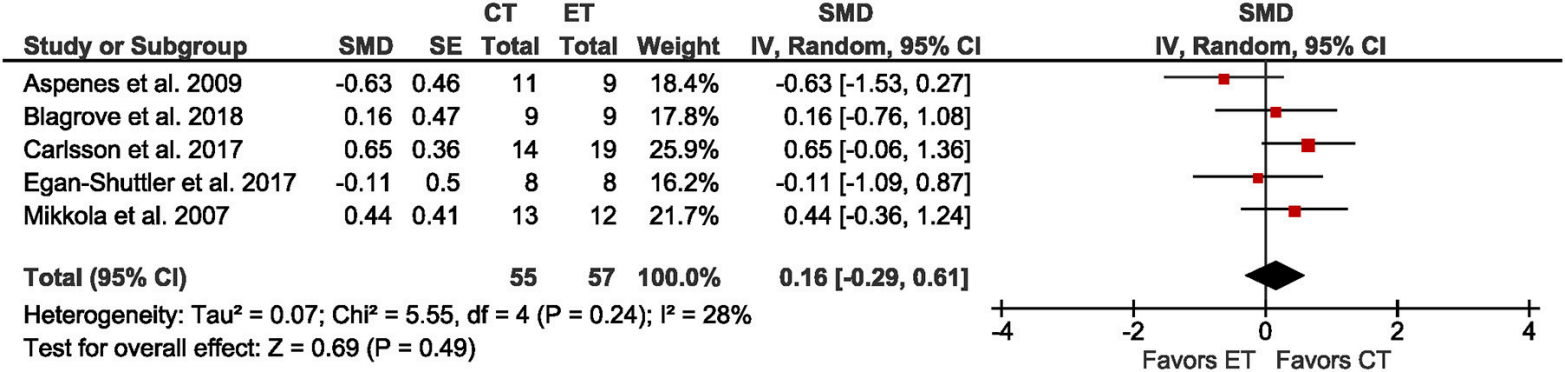
Forest plot for the outcome exercise economy in the comparison between singular endurance training (ET) and concurrent strength and endurance training (CT).

**Figure 4:**
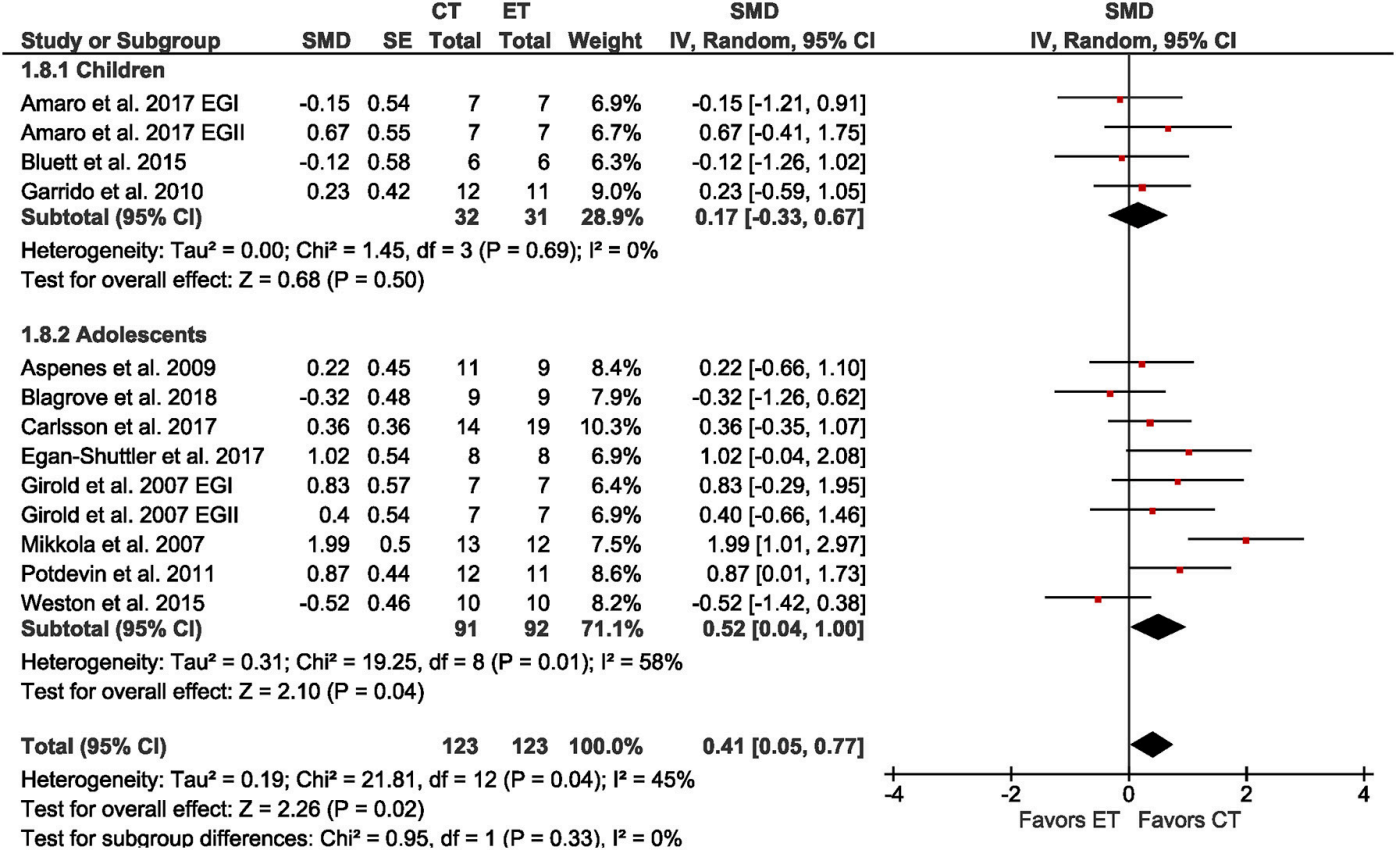
Forest plot for the outcome athletic performance in the comparison between singular endurance training (ET) and concurrent strength and endurance training (CT).

The meta-analytical comparisons between CT vs. ST and CT vs. ET are presented in the forest plots (Figures 2–4, 6) and are described in the next sections. Comparisons between CT and ET involved only young endurance athletes and comparisons between CT and ST involved non-athletic youth only.

### Effects of CT vs. ET on endurance-related outcomes

Four studies reported a measure of cardiorespiratory endurance, either as peak V°O_2_, or as V°O_2_max (Figure 2). Compared to ET, CT had a trivial effect on cardiorespiratory endurance in adolescent endurance athletes age 15.9 to 18.2 years (SMD = 0.04; *p* = 0.86; *I*^2^ = 22%). Due to the limited data, sub-group comparisons (trained vs. untrained, children vs. adolescents, boys vs. girls) were not possible.

Five studies reported exercise economy as V°O_2_ at a submaximal intensity during swimming, running, skiing on a treadmill, or rowing ergometry in adolescent athletes. The meta-analysis (Figure 3) revealed a trivial non-significant effect size (SMD = 0.16; *p* = 0.24; *I*^2^ = 28%) of CT compared to ET. Due to small sample sizes, sub-group analyses by age, sex, and training status were not possible.

All eleven studies involving young athletes reported a measure of athletic performance as the time of, or the mean velocity during, a time trial with distances ranging from 30 to 3000 m (median = 275 m). The meta-analysis (Figure 4) showed a significant small effect (SMD = 0.41; *p* = 0.02; *I*^2^ = 45%) of CT over ET. A sub-analysis of age revealed a moderate effect of CT over ET in adolescent athletes (SMD = 0.52; *p* = 0.02; *I*^2^ = 58%), but only a trivial effect in child athletes (SMD = 0.17; *p* = 0.50; *I*^2^ = 0%). However, the difference in effect sizes was not significant (χ^2^ = 0.95; *df* = 1; *p* = 0.33). Due to small sample sizes, sub-group analyses by sex and training status were not possible.

**Figure 5:**
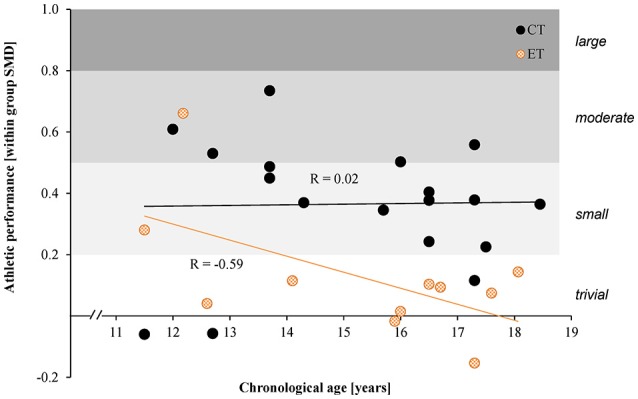
Scatterplot displaying the relationship between chronological age (y-axis) and within SMDs in athletic performance. Each dot represents one concurrent training (CT) or endurance training (ET) group.

We found a moderate negative association between chronological age and within group SMDs in athletic performance in young athletes following ET (*r* = −0.60; *p* = 0.04), but only a trivial correlation for CT (*r* = 0.02; *p* = 0.94) (Figure 5). In addition to the 11 studies in young endurance athletes we included data from two studies on CT in young soccer players (Enright et al., 2015; Makhlouf et al., 2016) to better be able to evaluate the relationship.

### Effects of CT vs. ST on strength/power-related outcomes

**Figure 6:**
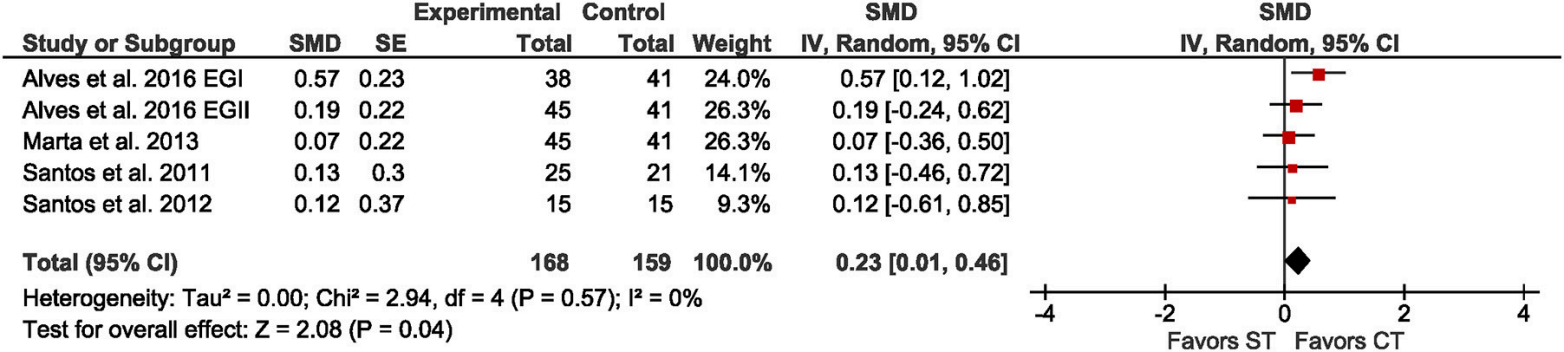
Forest plot for the outcome muscle power in the comparison between single-mode strength training (ST) and concurrent strength and endurance training (CT).

Four studies assessed vertical jump height (CMJ) as a proxy for lower extremity muscle power in non-athletic youth age 10.7 to 13.5 years. The meta-analysis (Figure 6) revealed a significant but small overall effect size of CT over ST (SMD = 0.23; *p* = 0.04; *I*^2^ = 0%). A sub-analysis did not reveal differences (χ^2^ = 0.14; *df* = 1; *p* = 0.71) between SMDs for children (SMD = 0.25; *p* = 0.04; *I*^2^ = 0%) and adolescents (SMD = 0.14; *p* = 0.66; *I*^2^ = n/a). Due to small sample sizes, sub-group analyses by sex and training status were not possible. No studies reported maximum muscle strength or muscle hypertrophy as outcomes.

## Discussion

This is the first systematic review that quantified the effects of CT vs. single-mode training (ST, ET) on selected measures of physical fitness and athletic performance in youth. We compared the effects of ET with CT on cardiorespiratory endurance, exercise economy, and athletic performance. We also compared the effects of ST with CT on leg power. CT resulted in larger improvements than single-mode ET on measures of athletic performance, whereas CT compared with ST improved leg power more. Due to small sample sizes, sub-group analyses by age, sex, and training status were not possible. As all comparisons between CT and ET were conducted in young endurance athletes and all comparisons between CT and ST included non-athletic youth, we address the groups in the discussion according to their training status.

### Effects of CT vs. ET in young endurance athletes

We hypothesized that CT is more effective than ET for improving athletic performance as assessed by time trials in young endurance athletes because muscle strength is a determinant of athletic performance (Faigenbaum et al., 2016). CT was more effective than single-mode ET to improve athletic performance assessed by time trials (Figure 4, SMD = 0.41, *p* = 0.04). This finding is in line with recommendations to include ST in the training of young athletes (Faigenbaum et al., 2009) and with models of long-term athletic development (Lloyd and Oliver, 2012) but direct evidence was limited to a handful of individual studies. Other meta-analyses incorporated young endurance athletes, but grouped them together with adult endurance athletes (e.g., Balsalobre-Fernández et al., 2016) or young athletes from different sports (Lesinski et al., 2016). Lesinski et al. (2016) also found moderate effects (SMD = 0.75) of ST on athletic performance in young athletes, mainly involving soccer players. As Lesinski et al. (2016), we also found low to moderate heterogeneity in outcomes of athletic performance, which suggests that the interpretation may be biased. Methodological differences between studies can increase heterogeneity: the distances of time trials ranged from 30 m to 3,000 m (median = 275 m) in running, swimming, and rowing. The moderate heterogeneity implies that the effectiveness of CT depends on distance and type of sport. The study revealing the largest effect of CT on athletic performance (SMD = 1.99) indeed evaluated athletic performance over the shortest distance (i.e., 30 m) in running (Mikkola et al., 2007). Whereas the study using the longest distance (i.e., 3,000 m) in running showed only a trivial effect (SMD = −0.12) (Bluett et al., 2015).

Sub-group analysis based on chronological age indicated a trend toward higher effects of CT vs. ET on athletic performance in adolescent athletes (Figure 4, SMD_children_ = 0.17; SMD_adolescents_ = 0.52). The relationship between chronological age and athletic performance in the CT groups (Figure 5, *r* = 0.02) and ET groups (Figure 5, *r* = −0.59) suggests that with increasing age the effects of CT on athletic performance do not increase, but the effects of ET alone decrease. This is in line with Lloyd and Oliver's (Lloyd and Oliver, 2012) youth physical development model that recommends using ST throughout developmental stages. Furthermore, previous training experience plays a mediating role in the magnitude of training adaptation (Fleck and Dean, 1987; Fyfe and Loenneke, 2018). The discrepancy in associations between CT and ET could be explained by previous training experience. It could be argued that familiarity with the exercises in the ET groups increased with age, as the ET groups primarily followed their habitual training. As a result, younger athletes with less training experience could benefit more from ET than the older athletes with more experience. The novel exercises in the CT groups could induce adaptations even in older athletes.

Improvements in cardiorespiratory endurance, exercise economy, and performance at lactate threshold may all increase endurance performance (Rønnestad and Mujika, 2014). The limited and heterogeneous data in the present review made it difficult to determine how CT more than ET improved athletic performance in young endurance athletes. The present data suggest that neither cardiorespiratory endurance nor exercise economy improves following CT in young endurance athletes. Previous studies in adults (Aagaard and Andersen, 2010; Sunde et al., 2010; Rønnestad and Mujika, 2014; Balsalobre-Fernández et al., 2016; Denadai et al., 2017) suggested that CT may improve endurance performance by increasing exercise economy, without affecting cardiorespiratory endurance. Of all the studies reporting time trials, not even half of them reported measures on exercise economy or cardiorespiratory endurance (4–5 out of 11). Such paucity of data together with between-group differences at baseline, make it difficult to understand the mechanisms underlying the improvements in time trial performance.

In summary, adding ST to ET seemed to potentiate the effects produced by ET, as CT improved endurance athletes' endurance performance more than did ET. Such a potentiation effect may be greater in adolescents compared to children. However, it is unclear how CT leads to improved athletic performance in young endurance athletes.

### Effects of CT vs. ST in youth

We hypothesized that youth improve physical fitness more when performing CT compared to single-mode training and that the interference effect can be observed in adolescents but not in children because they lack the hypertrophic response to ST (Ozmun et al., 1994; Granacher et al., 2011). The studies that compared CT to ST in non-athletic children (Santos et al., 2012; Marta et al., 2013; Alves et al., 2016) and adolescents (Santos et al., 2011) revealed that CT improved proxies of muscle power slightly more (Figure 6, SMD = 0.23, *p* = 0.04).

Unlike in adults (Wilson et al., 2012), combining ST and ET into CT resulted in a potentiating instead of an interference effect on untrained children's leg power. Perhaps the training status played a role in this potentiating effect in non-athletic children and adolescents. According to Coffey and Hawley (2017), “untrained individuals have a greater capacity to activate the molecular machinery in muscle in response to contractile activity, because any overload stimulus induces large perturbations to cellular homeostasis regardless of the mode of exercise.” Accordingly, ET produced hypertrophy (Konopka and Harber, 2014) and ST increased oxidative capacity in untrained muscle (Tang et al., 2006). In line with this observation, the studies included in the present meta-analysis showed that ST improved estimated V°O_2_max in non-athletic youth (Δ between +0.3 and +1.6 mmol·ml^−1^·kg^−1^) (Santos et al., 2011, 2012; Marta et al., 2013; Alves et al., 2016) compared with passive control groups (Δ between −1.1 and +0.3 mmol·ml^−1^·kg^−1^). A second explanation may be related to the use of the 20 m shuttle run as an endurance outcome (Santos et al., 2011, 2012; Marta et al., 2013; Alves et al., 2016). These studies used the 20 m shuttle run test as an endurance exercise. The constant acceleration and deceleration of the center of mass could act as a stimulus for leg power measured in the form of jump performance.

The interference effect associated with CT increased with training volume (Rønnestad et al., 2012) and when the form of ET in CT was running in adults (Wilson et al., 2012). Sequencing order of ST and ET elements of CT may affect the magnitude of interference. Muscle hypertrophy might be compromised when ET is performed during the 18 h after ST (Baar, 2014). However, this hypothesis relies heavily on animal data. Meta-analyses of human data seem to favor the ST → ET sequence (Eddens et al., 2017; Murlasits et al., 2017). For instance, the ST → ET compared to the ET → ST sequence produced ~7% larger gains (*p* < 0.01) in 1RM squat in athletic and non-athletic adults age 18 to 65 y (Eddens et al., 2017). There were no favorable outcomes for either sequence in static strength, muscle hypertrophy or cardiorespiratory endurance. Data in adolescent soccer players (age 17 y) suggest that sequencing can affect improvements in maximum muscle strength, power, and hypertrophy (Enright et al., 2015). Unlike in adults, the ET → ST vs. the ST → ET sequence was favored in adolescent soccer players. However, one limitation potentially biasing this conclusion was that athletes in the ET → ST group had slightly longer recovery time (2 h) and their lunch between training sessions while the athletes in the ST → ET group had shorter recovery time (< 1 h) and a protein shake. Conflicting findings were observed in 13-year-old soccer players (Makhlouf et al., 2016), suggesting that the sequence of ET and ST did not affect improvements in strength-related outcomes in children. The findings of differential responses to sequencing in children and adolescents may be explained by our hypothesis that the interference effect of endurance exercise on strength development is age-dependent. However, it has to be noted that the studies on sequencing effects included no ST groups. It is therefore impossible to determine whether sequencing produced a potentiating or an interfering effect. The available data suggest that responses to sequencing are age-dependent but it is unclear whether this translates to the interference effect. Therefore, more research is needed to test the hypothesis that interfering effects of endurance exercise on strength adaptations are age-dependent and present in adolescent but not in child athletes.

In summary, CT can improve lower extremity muscle power more than ST in non-athletic youth. This finding is indicative of a potentiating effect of CT. Weak evidence in young athletes suggests that age is a factor to consider when manipulating the sequence of ET and ST. It remains inconclusive whether interfering effects of endurance exercise on strength adaptations are age-dependent in youth.

### Strengths and limitations

This is the first review with a meta-analysis to examine the effects of CT in youth with a specific focus on young athletes. The available data allowed us to examine the effects of training on the most relevant outcome for practitioners, namely athletic performance. Furthermore, our data provided some preliminary insights into the interference hypothesis in youth.

The available data from the literature concerning underlying physiological mechanisms such as measures of neuromuscular activity or exercise economy were limited. While there are indications that the responses to CT are greater in adult female compared with male athletes (Barnes et al., 2013), this remains unresolved in young athletes due to insufficient data. In addition, we were not able to clarify whether interference effects in strength adaptations are more pronounced in adolescents compared with children, again due to a lack of data. Moreover, our conclusions are limited because the included studies did not control for training volume between CT and single-mode ST or ET (but see Mikkola et al., 2007). Thus, the observed effects in favor of CT could also be the result of additional training volume. A final limitation was the “fair” methodological quality due to the difficulty in blinding athletes to intervention and investigators to participants' group assignment.

### Recommendations

Based on present and past data (Faigenbaum et al., 2016; Lesinski et al., 2016), we recommend that practitioners and coaches include both ST and ET to increase endurance performance in young athletes and to improve physical fitness in non-athletic youth. Both ET and CT could be effective to improve athletic performance in children. However, from a long-term athletic development perspective (Lloyd and Oliver, 2012), CT appears to be favored. CT allows youth to become familiar with ST and learn proper exercise technique from which they may profit at a later age. Coaches should also be aware that sequencing ET and ST within CT affects performance outcomes in young (postpubertal) adolescent athletes. Adhering to previous recommendations could help minimize interference effects (García-Pallarés and Izquierdo, 2011; Baar, 2014; Murlasits et al., 2017). The ST → ET sequence may produce the best results in adolescent athletes but the order does not seem to differentially affect training adaptations in children. This recommendation requires confirmation, as it was based on data from two studies examining young soccer players age 13 and 17 y.

Future studies on CT should control training volume so that any potentiating or interfering effect is not due to differences in training volume between groups. There is a need to report measures quantifying not only athletic performance but also measures that can help understand the underlying processes such as exercise economy or muscle hypertrophy. Biological age should always be reported given their relevance to training adaptations. Finally, data and statistical analyses should be reported separately for boys and girls so that any sex effect on training adaptations can be determined.

## Conclusions

The current systematic review and meta-analysis examined the effects of CT on outcomes of physical fitness and athletic performance in youth. We found at worst no interfering but perhaps a potentiating effect of CT compared with ST or ET alone in endurance athletes age 10 to 18 years and non-athletic youth age 10 to 13 years. A potentiating effect of CT was most visible in adolescent endurance athletes. Preliminary findings from this meta-analysis suggest that CT improves lower body power more than ST in non-athletic youth. This is in contrast to the adult literature and implies an age-dependent interference effect of CT on measures of muscle power. When designing CT programs, training status, sequencing effects, and biological age are factors to consider in future studies.

## Author contributions

All authors listed have made a substantial, direct and intellectual contribution to the work, and approved it for publication.

### Conflict of interest statement

The authors declare that the research was conducted in the absence of any commercial or financial relationships that could be construed as a potential conflict of interest.
